# The changing use of the ovipositor in host shifts by ichneumonid ectoparasitoids of spiders (Hymenoptera, Ichneumonidae, Pimplinae)

**DOI:** 10.1051/parasite/2018011

**Published:** 2018-03-28

**Authors:** Keizo Takasuka, Niclas R. Fritzén, Yoshihiro Tanaka, Rikio Matsumoto, Kaoru Maeto, Mark R. Shaw

**Affiliations:** 1 Institute for Advanced Biosciences, Keio University, Yamagata Japan; 2 Graduate School of Agricultural Science, Kobe University, Hyogo Japan; 3 Zoological Museum, University of Turku, Turku Finland; 4 Koto-ku, Tokyo Japan; 5 Osaka Museum of Natural History, Osaka Japan; 6 National Museums of Scotland, Edinburgh UK

**Keywords:** Polysphinctine, Oviposition, Ventral-press, Dorsal-press, RTA-clade, Araneoidea

## Abstract

Accurate egg placement into or onto a living host is an essential ability for many parasitoids, and changes in associated phenotypes, such as ovipositor morphology and behaviour, correlate with significant host shifts. Here, we report that in the ichneumonid group of koinobiont spider-ectoparasitoids (“polysphinctines”), several putatively ancestral taxa (clade I here), parasitic on ground-dwelling RTA-spiders (a group characterised by retrolateral tibial apophysis on male palpal tibiae), lay their eggs in a specific way. They tightly bend their metasoma above the spider’s cephalothorax, touching the carapace with the dorsal side of the ovipositor apically (“dorsal-press”). The egg slips out from the middle part of the ventral side of the ovipositor and moves towards its apex with the parted lower valves acting as rails. Deposition occurs as the parasitoid draws the ovipositor backwards from under the egg. Oviposition upon the tough carapace of the cephalothorax, presumably less palatable than the abdomen, is conserved in these taxa, and presumed adaptive through avoiding physical damage to the developing parasitoid. This specific way of oviposition is reversed in the putatively derived clade of polysphinctines (clade II here) parasitic on Araneoidea spiders with aerial webs, which is already known. They bend their metasoma along the spider’s abdomen, grasping the abdomen with their fore/mid legs, pressing the ventral tip of the metasoma and the lower valves of the ovipositor against the abdomen (“ventral-press”). The egg is expelled through an expansion of the lower valves, which is developed only in this clade and evident in most species, onto the softer and presumably more nutritious abdomen.

## Abstract (fr)

Le placement précis des œufs dans ou sur un hôte vivant est une capacité essentielle pour de nombreux parasitoïdes, et les changements dans les phénotypes associés, tels que morphologie des ovipositeurs et comportement, sont corrélés avec des changements significatifs d’hôte. Nous rapportons ici que dans le groupe des ichneumonidés koinobiontes ectoparasitoïdes d’araignées (« polysphinctines »), plusieurs taxons putatifs ancestraux (clade I ici), parasites d’araignées terrestres RTA (un groupe caractérisé par une apophyse tibiale rétrolatérale sur les tibias des palpes mâles), pondent leurs oeufs d’une manière spécifique ; ils courbent étroitement leur métasome au-dessus du céphalothorax de l’araignée, touchant apicalement la carapace avec le côté dorsal de l’ovipositeur (« pression dorsale »). L’œuf s’écoule de la partie médiane du côté ventral de l’ovipositeur et se déplace vers son sommet avec les valves inférieures séparées agissant comme des rails, le dépôt de l’œuf se produisant lorsque le parasitoïde tire l’ovipositeur en arrière de sous l’œuf. La ponte sur la carapace dure du céphalothorax, vraisemblablement moins agréable au goût que l’abdomen, est conservée chez ces taxons, et présumée adaptative en évitant les dommages physiques au parasitoïde en développement. Cette voie spécifique de ponte est inversée dans le clade putatif dérivé des polysphinctines (clade II ici) parasites d’araignées Araneoidea avec des toiles aériennes, qui est déjà connu. Ils courbent leur métasome le long de l’abdomen de l’araignée, saisissent l’abdomen avec leurs pattes antérieures et médianes, pressent l’extrémité ventrale du métasome et les valves inférieures de l’ovipositeur contre l’abdomen (« pression ventrale »). L’œuf est expulsé par une expansion des valves inférieures, qui est développée seulement dans ce clade et évidente dans la plupart des espèces, sur l’abdomen, plus doux et probablement plus nutritif.

## Introduction

Oviposition is a crucial event in the life history of parasitoids [50] and placing the egg precisely into or onto a living host presents both behavioural and mechanical challenges. In addition to having a point sharp enough to pierce the integument of the host for the typical function of administering venom, the ovipositor in parasitoid wasps shows many structural adaptations for different functional behaviours [12,42,104,106,109], derived from the ovipositor’s base plan of an upper valve and a pair of more or less interlocking lower valves that can slide independently [105,107]. Apomorphic characters in its form can reflect phylogeny. In general, especially in the case of ichneumonoid ectoparasitoids of concealed hosts and endoparasitoids, the egg is expelled down a canal enclosed by the three valves to erupt near the ovipositor tip to reach an appropriate place for oviposition. However, in a few groups of ectoparasitoid ichneumonids with a koinobiont lifestyle (i.e. allowing the host to recover normal activity for a time after being attacked) in which the parasitoid can make direct bodily contact with the host, the egg may issue direct from the genital opening without involvement of the ovipositor, as is also seen in all aculeates which make similar contact with the food source of their offspring (briefly reviewed by Shaw & Wahl [114]).

**Figure 1:**
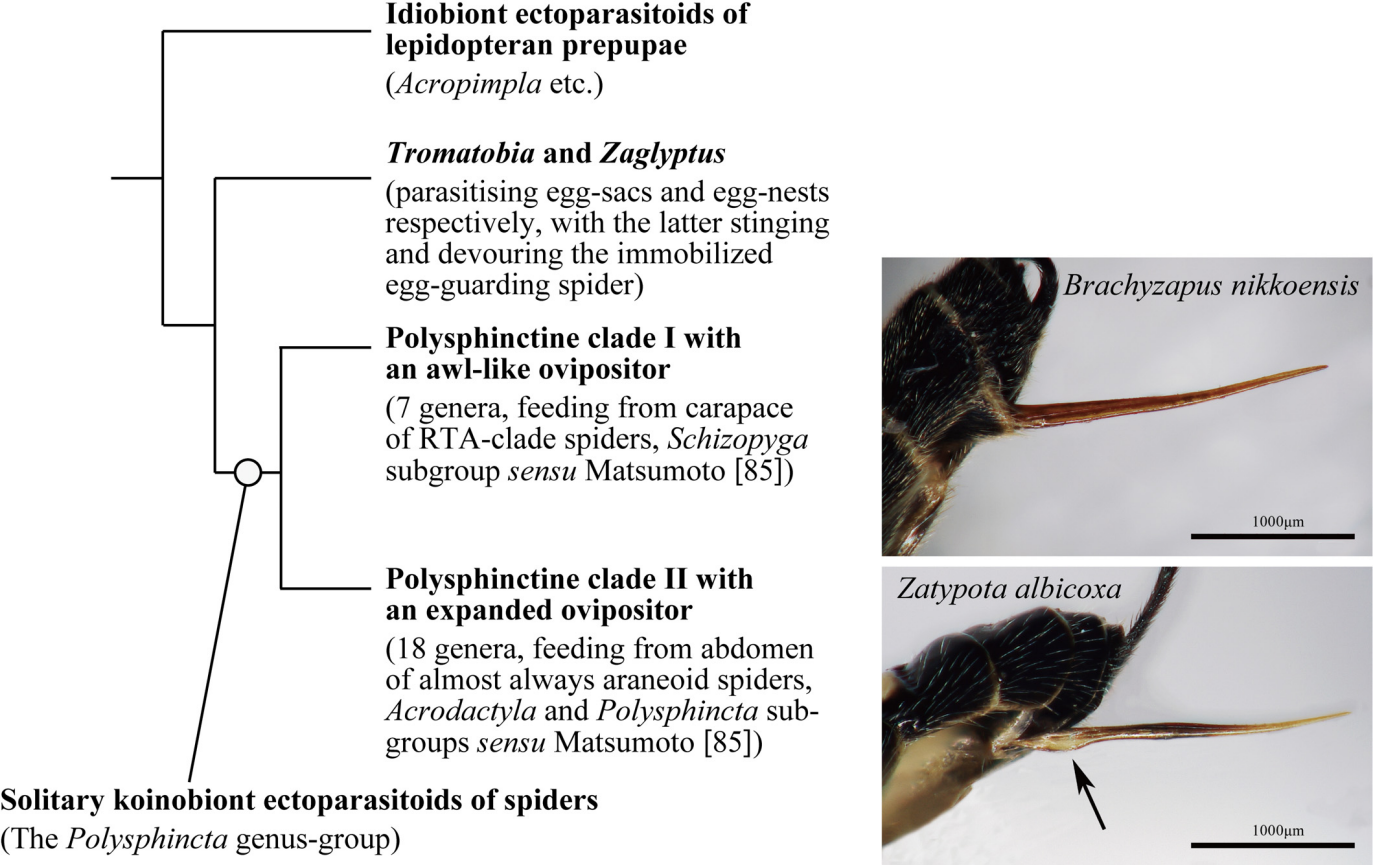
Simplified phylogenetic relationship within polysphinctine clades and its outgroups including spider egg-nest parasitoids, after Matsumoto [86]. The upper inserted photo is the awl-like ovipositor of *Brachyzapus nikkoensis*, a representative of clade I, and the lower one is the ovipositor of *Zatypota albicoxa*, a representative of clade II, with a ventral expansion at the proximal end of the lower valves (arrowed).

**Figure 2:**
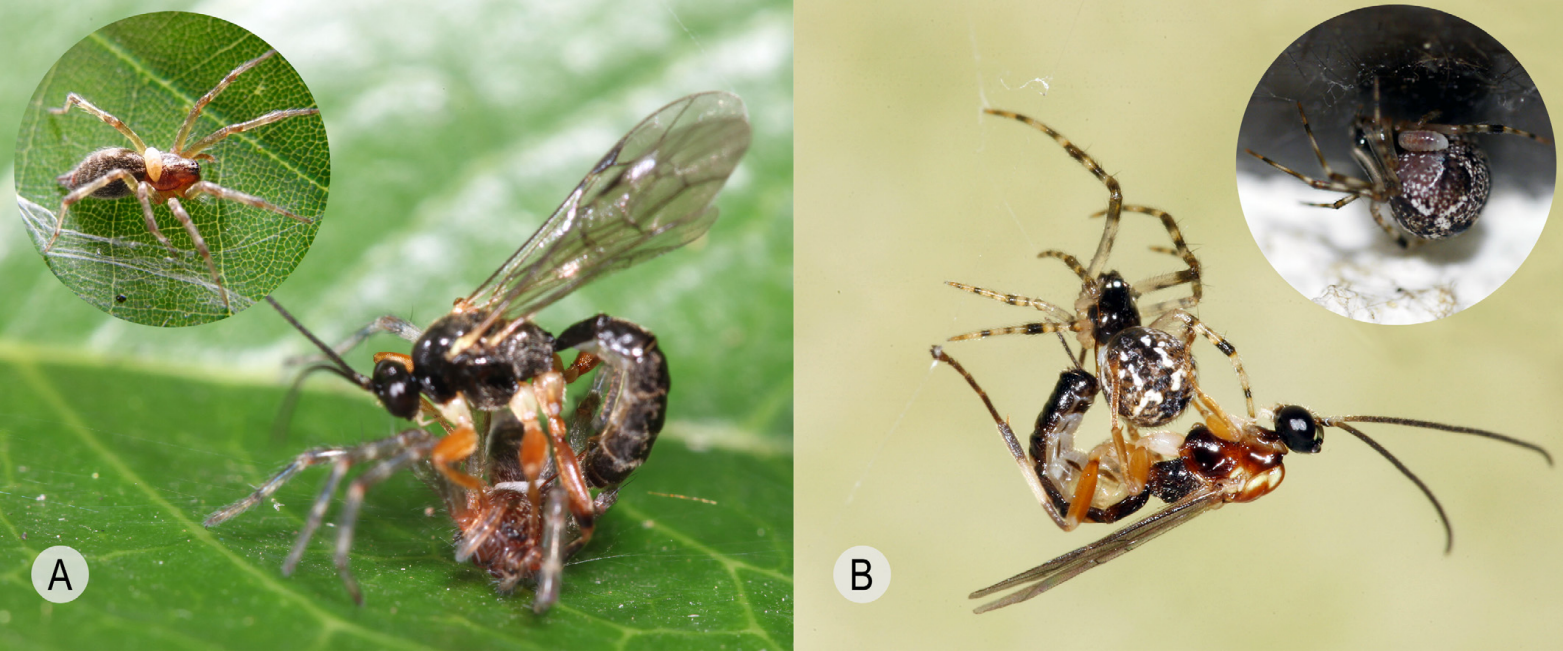
Oviposition by representative polysphinctines of clade I and II. **A** *Brachyzapus nikkoensis* (polysphinctine clade I) demonstrating the dorsal-press upon the cephalothorax of *Agelena silvatica* (Agelenidae), taken by Y.T. in Mizumoto Park, Tokyo, Japan in 14 June 2009. Circular inset is a middle instar larva of *B. nikkoensis* on the cephalothorax of *A. silvatica*. **B** *Zatypota albicoxa* (polysphinctine clade II) demonstrating the ventral-press upon the abdomen of *Parasteatoda tepidariorum* (Koch, 1841) [74] (Theridiidae), taken by Y.T. in Mizumoto Park, Tokyo, Japan in 6 August 2008. Circular inset is a middle instar larva of *Z. albicoxa* on the abdomen of *P. tepidariorum*.

Ichneumonidae, in general, parasitise the pre-adult stages of holometabolous insects, with a few groups having adopted spider egg-sacs and egg-nests as pabula, their larvae devouring successive eggs. In some cases (*Zaglyptus* in the subfamily Pimplinae), the maternal egg-guardian of cursorial spiders (e.g. Eutichuridae making egg-nests) is permanently immobilised and also consumed [70,95]. Probably from this association with egg-nests by certain Pimplinae (in addition to *Zaglyptus* there is *Tromatobia*, which oviposits into exposed undefended egg-sacs or defended ones without paralysis of the guarding spider), the *Polysphincta* genus-group (hereafter polysphinctines) has evolved as a unique lineage of solitary koinobiont ectoparasitoids of spiders [48,86,131] (Fig. 1; see also Fig. 2 insets), widely demonstrated to be monophyletic [48,86,108]. Polysphinctines inject venom in the host’s cephalothorax to cause temporarily paralysis for the purpose of oviposition, but after the egg is attached the spider recovers its normal activity until the parasitoid larva approaches full growth.

In a recent molecular study, Matsumoto [86] proposed two well-supported and reciprocally monophyletic major clades within this group: clade I, the *Schizopyga* subgroup *sensu* Matsumoto, and clade II equal to the *Acrodactyla* and *Polysphincta* subgroups *sensu* Matsumoto. Matsumoto [86] pointed out two ecological differences between the two clades: 1) they use different hosts and 2) the oviposition and hence larval development site on the host spider differs between the clades; larvae of clade I are exclusively attached to the cephalothorax while larvae of clade II are exclusively attached to the abdomen.

Matsumoto [86] regarded clade I as the more ancestral, with clade II being derived, and we outline our reasons for concurring with that view in the Discussion. However, the supposition that clade II is derived suggests of course that, if the polysphinctines are monophyletic as has been widely supported [48,86,108], the group called clade I will be paraphyletic with respect to clade II. In this paper, the relationship between the two supposed clades cannot be further elucidated, and “clade I” is used as a convenience term without the strict implication of unequivocal monophyly, though clade II is considered monophyletic.

For this paper, we have focused particularly on the behaviour of the group considered more ancestral (herein clade I), whose biology was previously poorly understood and for some groups unknown. Our detailed observations on the oviposition behaviour in both previously unstudied and already studied wasp genera strongly support the hypothesis proposed by Matsumoto [86] and we can show that the host groups used by the two wasp clades are not only ecologically but also phylogenetically different: clade I uses RTA-clade spiders (see Terminology) (hosts previously noted for three genera [36,37,85,86,95] and for a further two in this study), and clade II uses spiders of Araneoidea (numerous sources, see Table 1) with a single apparently secondary reversion (see Discussion). More importantly, we add a behavioural distinction regarding oviposition between the two clades, and additionally link differences in ovipositor morphology to the different behaviours.

**Table 1:** Behaviour and ecology (immature position and host taxa) in polysphinctine clades I and II. Note that *Hymenoepimecis robertsae* parasitising a nephiline spider is mentioned here from a drawing suggesting the dorsal-press as in clade I [35]. Owing to the lack of behavioural description in the paper, we believe further observation is needed before the stance it truly manifests can be determined. Nothing is known about the immature biology of *Dreisbachia, Inbioia* (clade I), *Aravenator*, *Chablisea*, *Lamnatibia*, *Pterinopus* and *Ticapimpla* (clade II) at present. Taxa in which oviposition stance has not been specified are grouped under the generic name.

Species	Matsumoto’s clade	Oviposition stance	Egg/larval position	Family of host (web type)	Reference
*Brachyzapus nikkoensis* (Uchida, 1928) [135]	clade I	dorsal-press (n =3)	cephalothorax [69,85]	Agelenidae (plane funnel web)	this study in Japan
*Piogaster* sp.	clade I	dorsal-press (n =6)	cephalothorax [see also 113]	Salticidae (cursorial)	this study in Finland
*Zabrachypus* sp.	clade I	dorsal-press (n =4)	cephalothorax [see also 112]	Titanoecidae (ground-web)	this study in Europe
*Iania pictifrons* (Thomson, 1877) [129]	clade I	dorsal-press (n =1)	cephalothorax [36,37,113]	Clubionidae (cursorial)	this study in Finland
*Schizopyga circulator* (Panzer, 1800) [101]	clade I	dorsal-press	cephalothorax [also 36, 37]	Clubionidae (cursorial) [also 36, 37]	[86]
*Schizopyga frigida* Cresson,1870 [25]	clade I	unknown	cephalothorax	Clubionidae (cursorial)	[36,37]
*Schizopyga podagrica* Gravenhorst, 1829 [58]	clade I	unknown	cephalothorax	Eutichuridae (cursorial)	[95]
*Reclinervellus tuberculatus* (Uchida, 1932) [136]	clade II	ventral-press	abdomen	Araneidae (orb web)	[87]
*Hymenoepimecis argyraphaga* Gauld, 2000 [48]	clade II	ventral-press	abdomen	Tetragnathidae (orb web)	[30]
*Hymenoepimecis veranii* Loffredo & Penteado-Dias, 2009 [83]	clade II	ventral-press	abdomen	Araneidae (orb web)	[53]
*Hymenoepimecis* *robertsae* Gauld, 1991 [46]	clade II	unclear which stance is used	abdomen	Araneidae, Nephilinae (orb web)	[35]
*Zatypota albicoxa*	clade II	ventral-press	abdomen	Theridiidae (cobweb)	[127]
*Zatypota maculata* Matsumoto & Takasuka, 2010 [88]	clade II	ventral-press	abdomen [88]	Theridiidae (cobweb)	K. Takasuka (in prep.) in Japan
*Acrodactyla carinator* (Aubert, 1965) [3], *A. degener* (Haliday, 1838) [62], *A. quadrisculpta* (Gravenhorst, 1820) [57]	clade II	unknown	abdomen	Tetragnathidae (orb web); Linyphiidae (3D web with a sheet)	[11,19,36,68,77,86,89]
*Acrotaphus* *fuscipennis* (Cresson, 1865) [24], *A. tibialis* (Cameron, 1886) [20], *A. williti* (Cresson, 1870) [25]	clade II	unknown	abdomen	Araneidae (orb web)	[16,33,55]
*Eriostethus minimus* Gauld, 1984 [45], *E. rufus* (Uchida, 1932) [136], *E. perkinsi* (Baltazar, 1964) [6]	clade II	unknown	abdomen	Araneidae (orb web), Theridiidae (cobweb)	[76,86]
*Eruga* cf. *gutfreundi* Gauld, 1991 [46], *E. telljohanni* Gauld, 1991 [46], *E. unilabiana* Pádua & Sobczak, 2017 [120]	clade II	unknown	abdomen	Tetragnathidae (orb web), Linyphiidae (3D web with a sheet)	[8,33,120]
*Flacopimpla barathrica* Fritzén, 2014 [40], *F. parva* (Cresson, 1870) [25], *F. varelae* Gauld, 1991 [46]	clade II	unknown	abdomen	Theridiidae (cobweb)	[27,40,121]
*Hymenoepimecis bicolor* (Brullé, 1846) [18], *H. japi* Sobczak, Loffredo, Penteado-Dias, & Gonzaga, 2009 [117], *H. jordanensis* Loffredo & Penteado-Dias, 2009 [83], *H. manauara* Pádua & Oliveira, 2016 [98], *H. neotropica* (Brues & Richardson, 1913) [17], *H. silvanae* Loffredo & Penteado-Dias, 2009 [83], *H. sooretama *Sobczak, Loffredo, Penteado-Dias, & Gonzaga, 2009 [117], *H. tedfordi* Gauld, 1991 [46]	clade II	unknown	abdomen	Araneidae (orb web); Araneidae, Nephilinae (orb web); Araneidae, Cyrtophorinae (3D web with a dome-shape platform); Tetragnathidae (orb web)	[33,52,54,98,115–117]
*Longitibia* sp.	clade II	unknown	abdomen	Linyphiidae (3D web with a sheet)	R. Matsumoto and K. Takasuka (unpublished) in Japan
*Megaetaira madida* (Haliday, 1838) [62], *M. varicarinata* (Uchida & Momoi, 1958) [138]	clade II	unknown	abdomen	Tetragnathidae (orb web)	[36,63,92]
*Oxyrrhexis carbonator* (Gravenhorst, 1807) [56], *O. carbonator texana* (Cresson, 1870) [25], *O. zephyrus* Fritzén, 2014 [41]	clade II	unknown	abdomen	Theridiidae (cobweb)	[26,41]
*Polysphincta boops* Tschek, 1869 [133], *P. gutfreundi* Gauld, 1991 [46], *P. janzeni* Gauld, 1991 [46], *P. koebelei* Howard, 1892 [67], *P. longa* Kasparyan, 1976 [71], *P.* sp. nr. *purcelli* Gauld, 1991 [46], *P. rufipes* Gravenhorst, 1829 [58], *P. tuberosa* Gravenhorst, 1829 [58]	clade II	unknown	abdomen	Araneidae (orb web)	[7,14,32,36,43,72,73,78,111]
*Reclinervellus masumotoi* Matsumoto & Konishi, 2007 [87], *R. nielseni* (Roman, 1923) [110]	clade II	unknown	abdomen	Araneidae (orb web)	[38,69,87,124,128]
*Sinarachna nigricornis* (Holmgren, 1860) [65], *S. pallipes* (Holmgren, 1860) [65]	clade II	unknown	abdomen	Araneidae (orb web)	[36,37,93]
*Zatypota* spp. (numerous)	clade II	unknown	abdomen	Theridiidae (cobweb); Linyphiidae (3D web with a sheet); Dictynidae (cobweb); Araneidae (orb web)	[7,9,31,39,40,51,66,67,75,79,80,88,119,124,140,142]

## Terminology

*Cephalothorax*: The fused head and thorax of spiders, also called the prosoma.

*Carapace*: A sclerotized plate covering the cephalothorax dorsally.

*RTA-clade*: A huge monophyletic lineage of spiders which was first proposed by Coddington and Levi, having retrolateral tibial apophysis (hence RTA) on male palpal tibiae [23]. Subsequently RTA has been consistently recognised to be a clear major (clade-defining) synapomorphy in many phylogenetic studies of Araneae [1,13,22,44,59,60,90,122,143]. The RTA-clade currently consists of 43 families in eight higher groups in accordance with Wheeler *et al.* [143], which are largely vagabonds or ground web weavers. The superfamily Titanoecoidea has been proposed to be an immediate outgroup of the RTA-clade [60,143].

## Materials and methods

We observed oviposition behaviour of several European and Japanese polysphinctine species *in vitro* and in the field, parts of which will be published in detail later. *Zatypota albicoxa* (Walker, 1874) [141], a member of clade II, for which oviposition behaviour is already known [125–127], was also looked at in more detail for corroboration and comparison with clade I. We focused especially on how the wasps use their ovipositor when ovipositing. The known behaviour of polysphinctines from published sources (mainly clade II), together with our new findings, are listed in Table 1 (Oviposition stance). In addition to behaviour, data on egg position (Egg/larval position) and host family records with their web type in parenthesis (Family of host) given in Table 1 has been gleaned from the literature when either description or illustration has been unequivocal.

For data on ovipositor morphology, we largely follow Gauld & Dubois [48], but we have personally checked species of most of the polysphinctine genera, particularly focusing on species and literature concerning genera where our observations on ovipositor morphology differ from that indicated in Gauld & Dubois [48] (*Oxyrrhexis*) or for genera of particular interest for their ovipositor morphology (e.g. *Oxyrrhexis* and *Chablisea*).

## Results

### Oviposition stance

**Figure 3:**
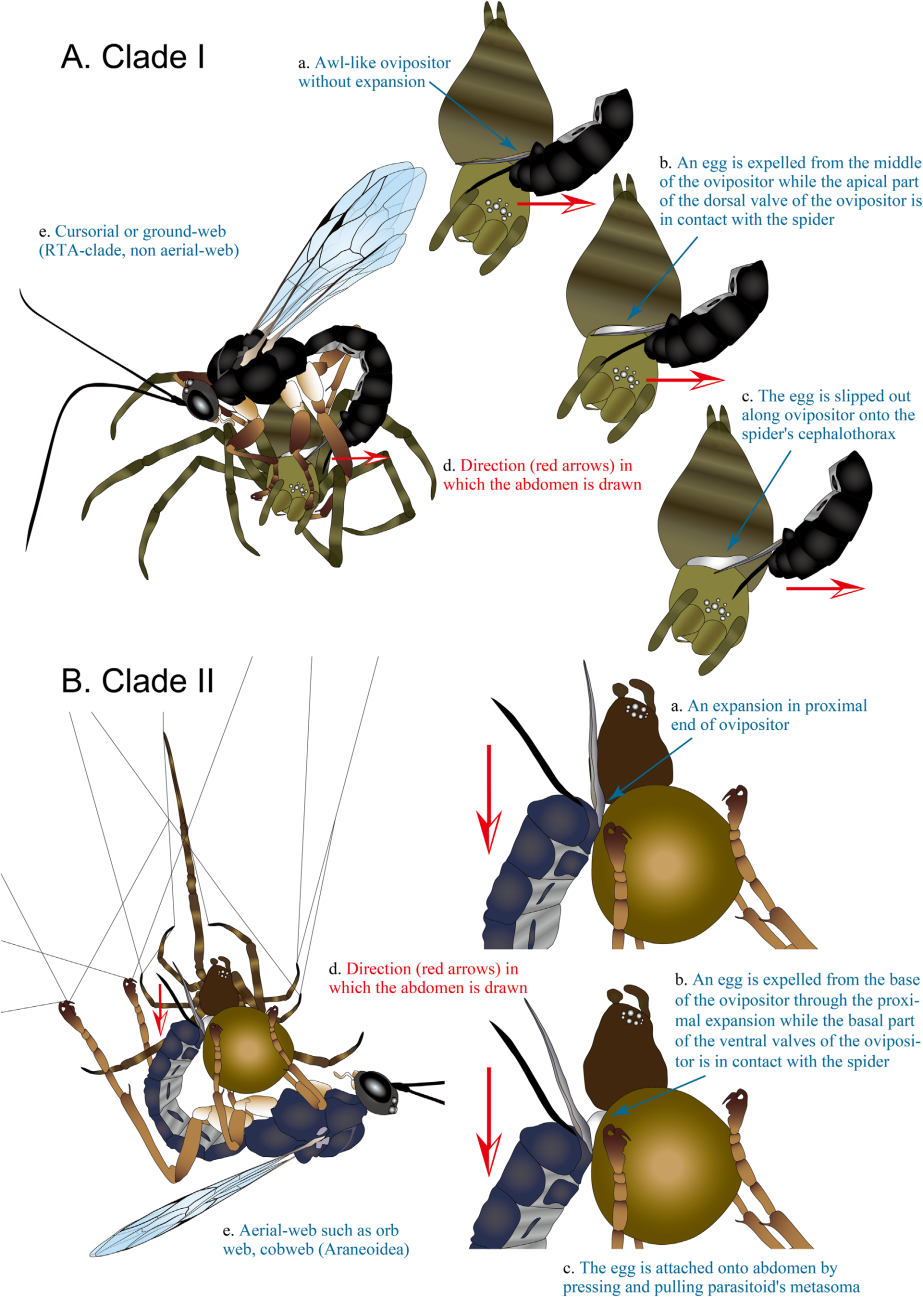
Schematic diagram of oviposition behaviour and egg-expulsion. **A** the dorsal-press of clade I, **B** the ventral-press of clade II. Lowercase symbols are explanations of characteristics that differ between the two clades.

We first discovered that *Brachyzapus nikkoensis*, belonging to clade I of the polysphinctines, lays its egg on the host spider (3 observations in total by 2 individuals in Tokyo, Japan in June 2009, host *Agelena silvatica* Oliger, 1983 [97], Agelenidae) in a rather specific way, contrasting with what we had seen in other genera (belonging to clade II). After temporarily paralysing the spider by stinging into its cephalothorax, the wasp initially bends its metasoma inward underneath its body with the dorsal tip of its metasoma and, in particular, the dorsal side of the ovipositor’s apical part touching the spider’s carapace (dorsal-press, Figs. 2A, 3A-d, S1). It then stretches the metasoma out (Fig. 3A-d, S1) while the egg is expelled onto the spider’s carapace. When doing this, the wasp grasps the host’s cephalothorax with its legs, having its head and ovipositor both pointing in the same direction, and the wasp thus has visibility of the oviposition site. Later, our studies on members of a further three genera, *Piogaster* sp. (cf. *pilosator* Aubert, 1958 [2]) in Finland in 2015 and 2016 (6 observations in total by 2 individuals, host *Salticus cingulatus* (Panzer, 1797) [100], Salticidae), *Zabrachypus* sp. from Germany ex *Titanoeca quadriguttata* (Hahn, 1833) [61] in 2015 (4 observations in total by 3 individuals, surrogate Finnish host *Titanoeca spominima* (Taczanowski, 1866) [123], Titanoecidae) and *Iania pictifrons* in Finland in 2016 (1 observation, host *Clubiona subsultans* Thorell, 1875 [130], Clubionidae), showed that this behaviour was the common one for clade I. Additionally, Matsumoto [86] gives a photograph of *Schizopyga circulator* clearly ovipositing on *Clubiona rostrata* Paik, 1985 [99] (Clubionidae) in the same way. In ovipositions by *B. nikkoensis* studied *in situ* and *Piogaster* sp. studied *in vitro*, the subjugated host lay prone (the ventral side below) on the plane sheet web or the substrate with the parasitoid sitting astride. Concerning *I. pictifrons* and *Zabrachypus* sp. studied *in vitro*, at least occasionally the temporarily paralysed spider can be hanging from its silk with the parasitoid almost being upside down but still employing the typical dorsal-press action and ovipositing onto the carapace.

For the species in clade II, the movement of the metasoma during oviposition is very different. The female presses the ventral tip of its metasoma against the spider, making contact with the ventral side of at least the base of the ovipositor, and pulls the metasoma inward (ventral-press, Figs. 2B, 3B-d, S2), while the egg is expelled onto the spider’s abdomen. When doing this, the wasp uses its legs more widely to grasp the abdomen and surrounds, and the head of the wasp is far away from the oviposition site (on small host specimens it may even be on the opposite side of the host) with the ovipositor tip and head pointing in opposite directions. Thus, it has little or no visibility of the oviposition site.

### Oviposition site

In clade I, the egg is applied exclusively to the carapace of the host’s cephalothorax (Figs. 2A, 3A-c) (with our two additions, *Zabrachypus* and *Piogaster*, it is recorded in altogether five of the seven genera of clade I, Table 1) whereas in clade II it is attached exclusively to the abdomen of the host (Figs. 2B, 3B-c), corroborated by many sources without exception (observed in 13 out of the 18 genera of clade II, Table 1).

###  Egg expulsion

At least in *B. nikkoensis* (n = 3) and *Piogaster* sp. (n = 1) (less complete observations on *I. pictifrons* (n = 1), and *Zabrachypus* sp. (n = 1) are consistent but inexact) the egg issues from the middle part of the ventral side of the ovipositor (Fig. 3A-b) and is guided distally along the ovipositor with the parted lower valves acting as rails (Fig. 3A-c), finally being placed onto the carapace as the ovipositor is withdrawn backwards from under it. In the species of clade II that we have studied, the egg is expelled neither from the middle region of the ovipositor nor from the genital opening but from near the base of the ovipositor (Figs. 2B, 3B-b). Egg expulsion ensues here at the site of an expansion of the lower valves of the ovipositor (Figs. 1 arrow, 3B-a). During oviposition in both dorsal-press and ventral-press behaviours, the ovipositor itself is finally withdrawn in the same overall direction in relation to the egg, while the egg is placed (S1, S2).

### Ovipositor morphology of clades I and II

The base of the ovipositor is ventrally simple (Figs. 1  upper inset, 3A-a, 4) in all known genera of clade I, whereas in most genera of clade II, it is distinctly expanded (Figs. 1 lower inset, 3B-a, 5). Only in *Oxyrrhexis* (Fig. 6A), *Chablisea* (Fig. 6B) and *Aravenator kamijoi* Momoi, 1973 [91] (Fig. 6C) of clade II is this expansion at least superficially lacking (see [41,82,102]). *Polysphincta* has a rather weak expansion (Fig. 6D) in comparison with other clade II genera.

**Figure 4:**
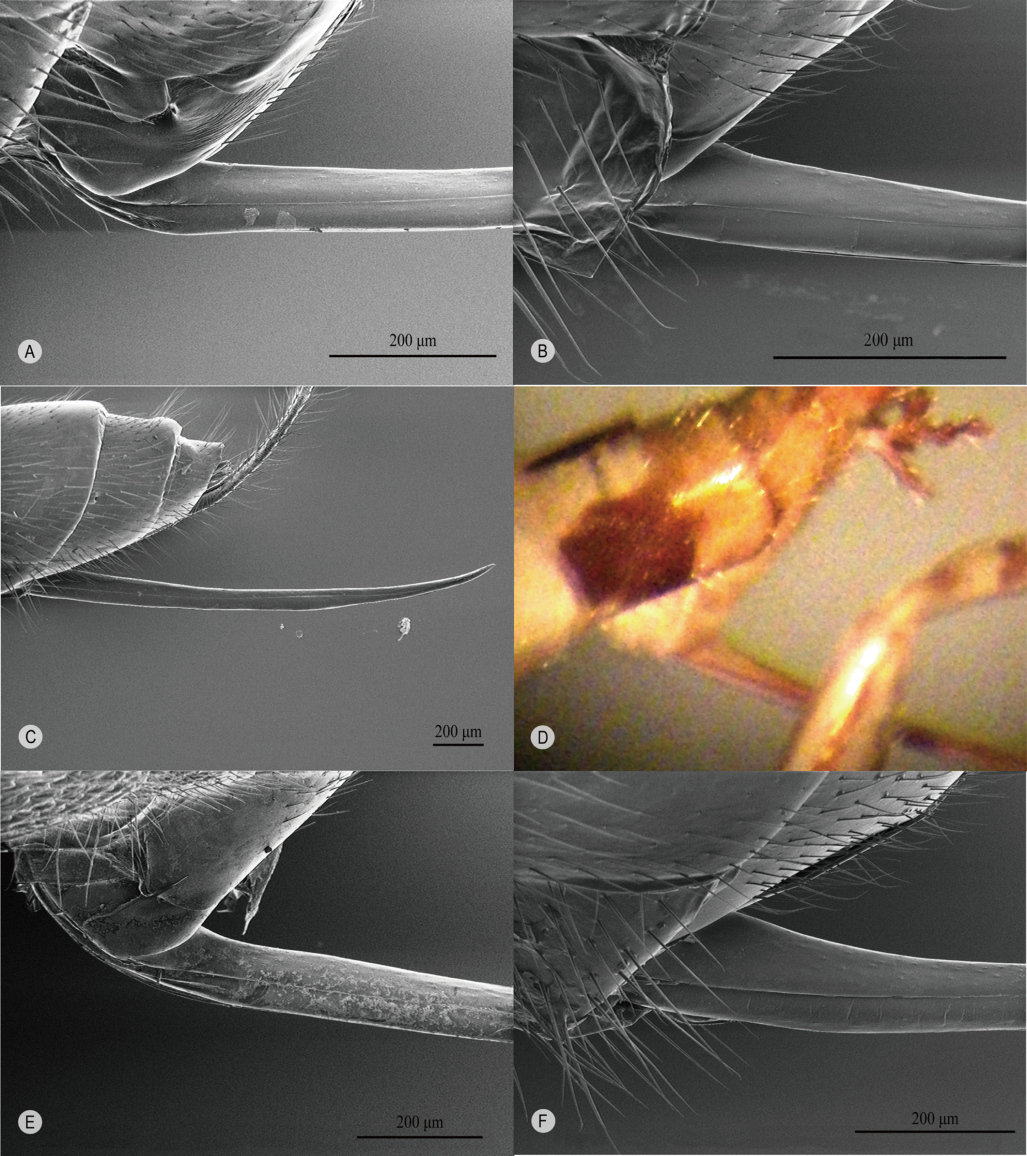
Awl-like ovipositor of clade I spp. with no ventral expansion at the proximal end. **A** *Brachyzapus nikkoensis*, **B** *Dreisbachia punctata* (Uchida & Momoi, 1959) [139], **C** *Iania* sp., **D** *Inbioia pivai* Gauld & Ugalde Gómez, 2002 [49] (holotype in NHM), **E** *Piogaster* *daisetsuzana* Kusigemati, 1985 [81], **F** *Schizopyga frigida*.

**Figure 6:**
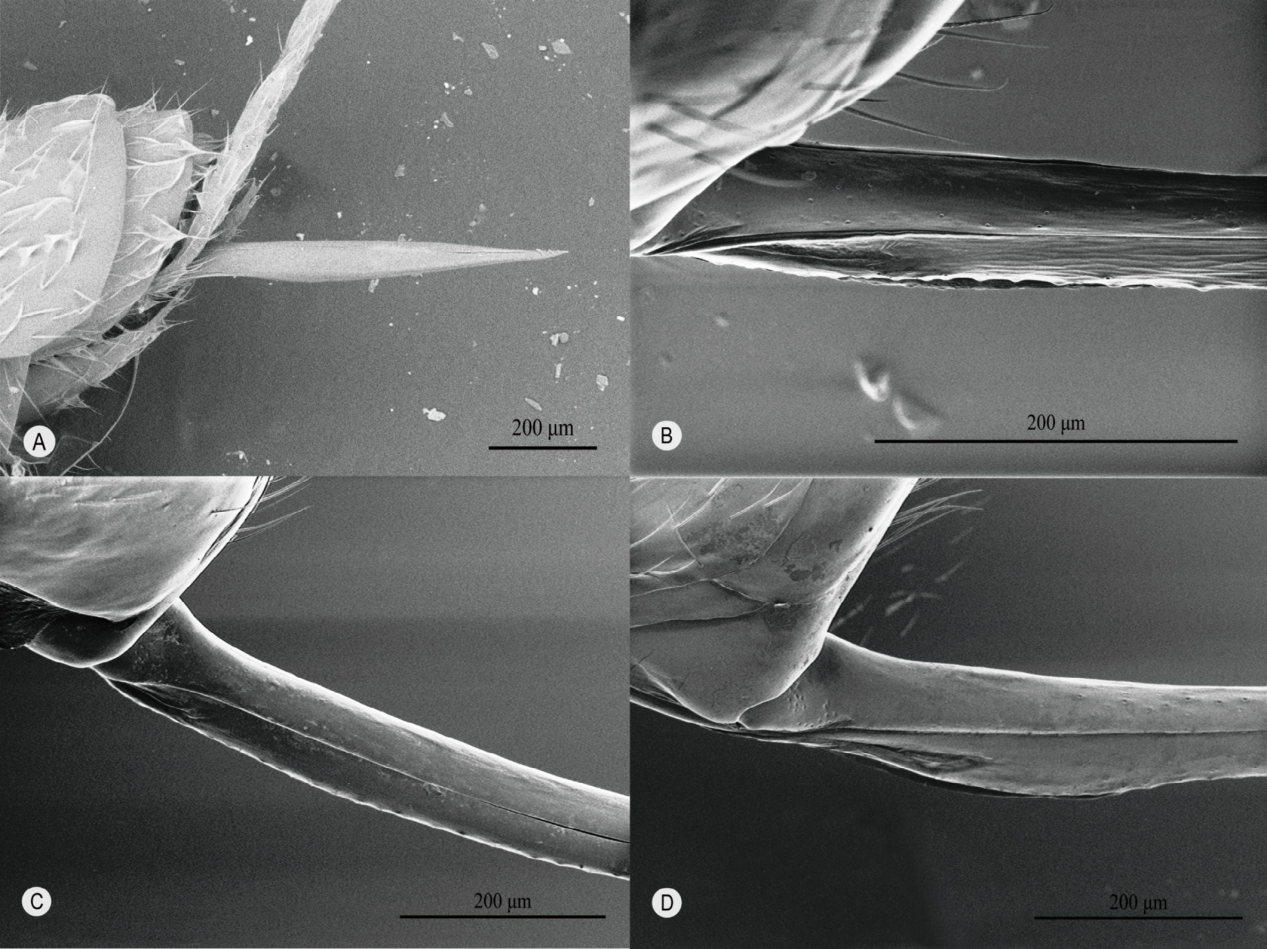
Ovipositor of clade II spp. with little or no expansion near its proximal end. **A** *Oxyrrhexis carbonator*, **B** *Chablisea* sp., **C** *Aravenator kamijoi*, **D** *Polysphincta rufipes*.

### Taxonomy of the hosts

When known hosts for the genera of clade I including the new hosts discovered by us (for *Zabrachypus* and *Piogaster*), and the hosts for clade II (Table 1) are mapped onto the most recent phylogeny of spiders [29,44,143], it is evident that the two polysphinctine clades use phylogenetically different groups of hosts. Clade I utilises both cursorial and ground-web building spiders exclusively of the RTA-clade (Figs. 2A, 3A-e), plus the superfamily Titanoecoidea constituting an immediate outgroup of the RTA-clade [143], while clade II taxa attack aerial-web weaving spiders, of which all but a few (Dictynidae, see Discussion) belong to the superfamily Araneoidea (Figs. 2B, 3B-e).

## Discussion

We have discovered that there are two clearly different oviposition stances in the polysphinctines, which we have called the dorsal-press and the ventral-press, reflecting which side of the ovipositor is in contact with the host. The former was previously unknown. We have also found that the different stances are linked to the phylogeny of the wasps, and additionally that the two stances clearly correlate with egg placement (and thus also larval development site), ovipositor morphology, place of egg expulsion, and finally with host taxonomy.

We posit that the dorsal-press, our newly-discovered oviposition stance used by polysphinctines of clade I only, which is the group regarded as the more ancestral [86], indicates an early evolutionary path of spider-ectoparasitism in comparison with that of clade II, in which species use the ventral-press.

The arguments advanced by Matsumoto [86] for regarding clade I as the more ancestral of the two major clades are accepted here, with some additional supporting observations. The genus *Tromatobia*, one of the immediate outgroups in Matsumoto’s analysis, mainly uses egg-sacs of Araneoidea [4,5,21,28,34,36,37,46,63,84,92,96,103,118,132,137] (more rarely other groups [4,36,37,64]) that are either unguarded or, if guarded, the parasitoid does not envenomate let alone consume the female spider. In fact, as far as is known, it has no adverse interaction with the spider itself. These Araneoidea do not have a role as hosts of clade I polysphinctines but are the almost invariable hosts of clade II polysphinctines. However, there seems little in the biology of *Tromatobia* to suggest that it is close to the ancestry of clade II polysphinctines. On the other hand, the main hosts of *Zaglyptus* (the other immediate outgroup in Matsumoto’s analysis) are in concealed egg-nests with a guarding female in RTA-clade families such as Clubionidae, Eutichuridae and Salticidae [4,34,36,37,69,70,92,95] (rarely using hosts outside the RTA-clade such as Araneidae, Theridiidae and Tetragnathidae [4,36,37]), and the female is not only stung and permanently immobilised prior to oviposition but also at least partly consumed by the ensuing progeny [70,95] and, perhaps most significantly, the guarding spider will be consumed if she is still gravid when paralysed [95]. Thus, the closest biology, host associations, and degree of physical contact exhibited by known taxa parasitising spider egg-sacs or egg-nests involves RTA-clade hosts which are attacked in more or less concealed sites. This strongly suggests that these hosts will be involved in the ancestry of the polysphinctines as a whole, such that clade I is likely to be the more ancestral of the two clades. Despite the finding by Matsumoto [86] that in his phylogeny the single included species of *Clistopyga* fell outside the clade representing the polysphinctines + the two genera included in his analysis (*Tromatobia* and *Zaglyptus*) that use spider egg-sacs or egg-nests, it seems possible that a reappraisal of the position of *Clistopyga* may help to elucidate the origin of the polysphinctines. In this case, clade I would again be indicated as the more ancestral. This is because one species of *Clistopyga* is known to be a solitary idiobiont ectoparasitoid of adult/subadult Salticidae (in the RTA-clade) alone in its nest [42], while others have been reared in small broods from concealed nests of the RTA-clade Clubionidae and the more ancestral [143] and biologically similar (ground and rock-fissure tube-web [10,15]) Segestriidae containing egg-sacs within which they appeared to have fed [36,94].

The dorsal-press of clade I is linked to the following: **1)** oviposition on the cephalothorax of the host (Fig. 2A), **2)** base of lower valves of the ovipositor simple (Figs. 1 upper inset, 3A-a, 4), **3)** egg expulsion along the middle part of the ventral side of the ovipositor (Fig. 3A-b), and **4)** utilisation of cursorial or ground-web weaving hosts belonging to the RTA-clade of spiders and its immediate outgroup (Table 1, Figs. 2A, 3A-e). The ventral-press of clade II is linked to **1)** oviposition on the abdomen of the host (Fig. 2B), **2)** expanded base of the lower valves of the ovipositor (Figs. 1 lower inset, 3B-a, 5), **3)** egg expulsion from the base of the ventral side of the ovipositor (Fig. 3B-b), and **4)** utilisation of aerial-web weaving hosts (Figs. 2B, 3B-e) that, with only a few exceptions, belong to the spider superfamily Araneoidea (Table 1).

**Figure 5:**
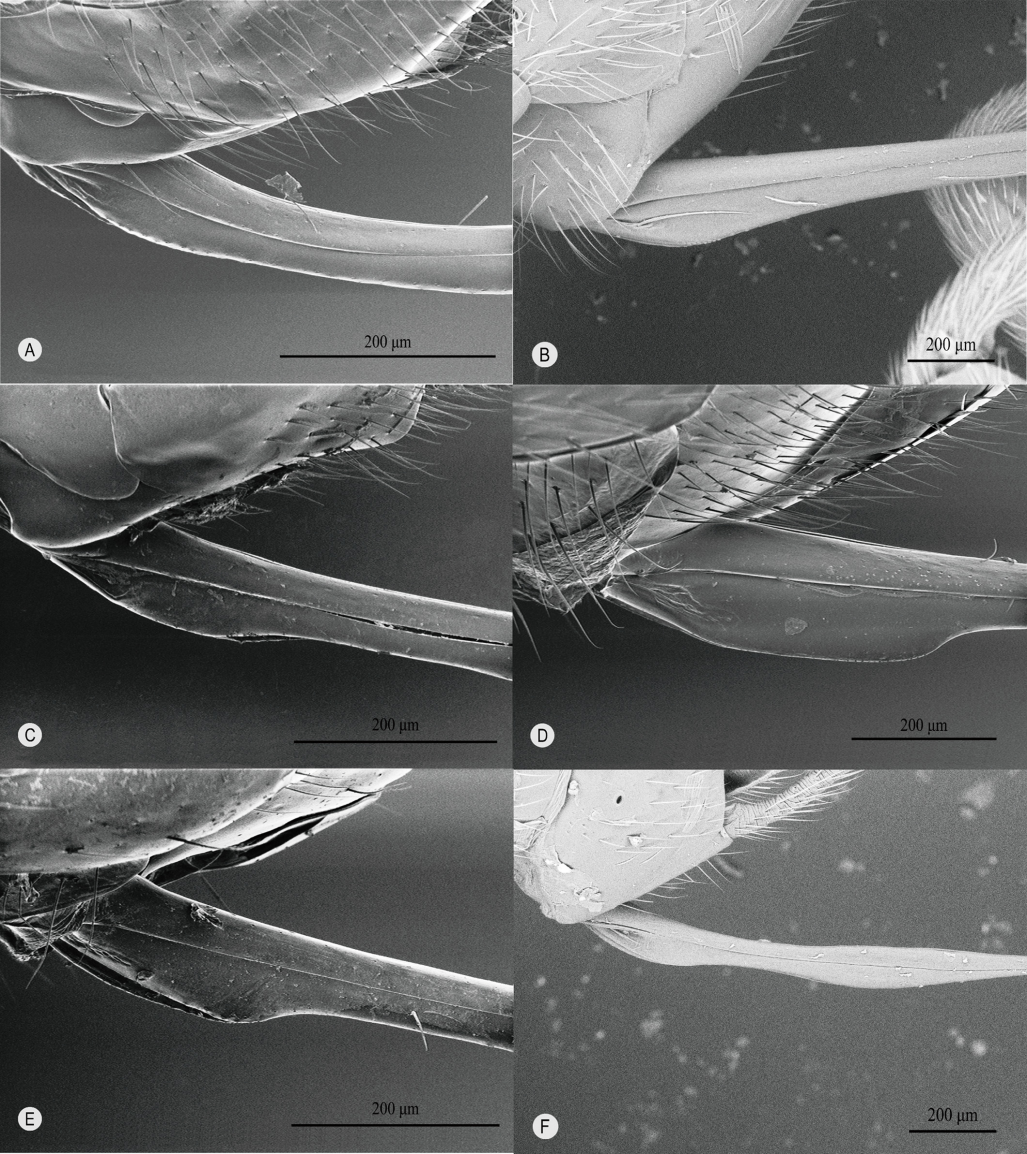
Ovipositor of clade II spp. with expansion near its proximal end. **A** *Acrodactyla takewakii* (Uchida, 1927) [134], **B** *Eriostethus rufus*, **C** *Megaetaira varicarinata*, **D** *Reclinervellus tuberculatus*, **E** *Sinarachna* sp., **F** *Zatypota maculata*.

The oviposition behaviour (as opposed to mere egg placement, which is very much better observed) for species of four genera in clade I, documented by us, and additionally *Schizopyga* illustrated by Matsumoto [86], has previously not been known. However, the oviposition behaviour in clade II has already been seen in three genera (Table 1). Additionally, the movement of the metasoma during oviposition in clade II had not been thoroughly described before.

Several species of clade II are already known not to expel the egg from the tip of the ovipositor (see [127]; Figs. 2B, 3B-b, S2). Although the eggs had previously been considered to be expelled from the genital opening (= below the base of the ovipositor, near the tip of metasoma) [30,87,127], we have seen in several species that they are actually expelled from the base of the ovipositor (Figs. 2B, 3B-b, S2). This is not easily seen and we thus suspect that the interpretations of previous observations could be wrong and that all members of clade II actually expel the egg from the base of the ovipositor, not from the genital opening (unlike e.g. Aculeata). Eggs are expelled from the expansion at the proximal end of the ovipositor’s lower valves (Fig. 3B-b), seemingly an autapomorphy of clade II, indicating a potential functional relationship between this modification and the ventral-pressing behaviour (Fig. 3B-c). For example, in whatever way it is mechanically involved in the expulsion of the egg and/or adhesive material, the expansion at the proximal end may have a sensory function facilitating exact egg placement or simply a role of steadying the ovipositor onto the spider’s skin while the egg is laid.

The egg expulsion along the middle part of the ovipositor reported here for members of clade I has not previously been documented in polysphinctines. We have clearly seen this in *B. nikkoensis* and in *Piogaster* sp., and our further incomplete observations (*I. pictifrons* and *Zabrachypus* sp.) suggest that it is likely to be the case in the other genera of clade I as well.

There are also noteworthy differences between the two clades ecologically. We can show that the two clades use phylogenetically different groups of hosts with partly distinctly different ecology. Clade I utilises both cursorial (hunting without a web but sometimes with a silken chamber for resting or egg-laying [cf. Fig. 3 in 15]) and ground-web building spiders exclusively of the RTA-clade and its immediate outgroup (Titanoecidae). Conversely, clade II attacks aerial-web weaving spiders mostly of the superfamily Araneoidea. These two spider groups form major distinct lineages within Entelegynae, which is hyperdiverse and the most derived monophyletic group, i.e. comprising the two major lineages (RTA-clade and Araneoidea) with several subclades [29,44,143].

Polysphinctine clades I and II apparently separated through a host shift, and many well-preserved characters are linked to oviposition and immature development. Parasitism of non-aerial-web weaving spiders, i.e. the RTA-clade (Figs. 2A, 3A-e), presumably practised by the first true polysphinctines, is evidently connected with the exclusive use of the cephalothorax as oviposition site where larval development commences, although after substantial growth and when the host is quiescent in its retreat, the larva also feeds from and consumes the abdomen [cf. Fig. 2a in 85]. Likewise the final instar larva of clade II also feeds from the cephalothorax of its recently killed host [30]. Despite the resulting larva then being faced with arguably a tougher pabulum than might be provided by the softer tissues of the host’s abdomen, one possible reason for the use of the cephalothorax instead of the abdomen in clade I is that the egg and larva would be more exposed to physical damage on the abdomen than in a lower position on the carapace of RTA-spiders actively running on the plane and hiding in small crevices or in a thin tubular retreat, even in RTA web-weavers (e.g. Agelenidae). An obvious general need for accuracy in egg deposition might be served well by using the orientation of the ovipositor over a substantial part of its length as a guiding device, adequately facilitating oviposition onto the relatively flat surface of the carapace. Also, visibility of the oviposition site (Fig. 2A) would be aided by the stance adopted for the dorsal-press.

On the other hand, aerial-web weaving host spiders used by clade II (mainly Araneoidea; Figs. 2B, 3B-e) usually settle in or on their webs, probably causing less risk of physical damage to a polysphinctine egg or larva by strenuous movement. This also opens up easier access to these softer and arguably more palatable or nutritious tissues for the larva. In addition, these spiders typically droop from their own threads after subjugation and the parasitoid hangs from the spider’s abdomen via its fore/middle legs (Figs. 2B, 3B). In such unstable situations, the ventral-press might facilitate egg deposition onto a distant and less visible side of the abdomen (Figs. 2B, 3B), with a consequent transfer of the site for egg expulsion and an expansion of the ovipositor at its proximal end.

Once the major host shift occurred between clades I and II, the two opposite oviposition stances seem to be largely fixed for coping with each host spider group. At least on the basis of the extant biota, the utilisation of Araneoidea appears to have promoted a stronger radiation and/or survival among clade II (18 genera, >200 species). This is probably associated with feeding advantages and easier location of the conspicuous webs of Araneoidea than is seen among clade I (7 genera, <50 species), despite the greater species-richness of the RTA-clade (24,681 species in 2,073 genera [144], 43 families of eight higher groups [143]) than Araneoidea (12,394 species in 1,130 genera [144], 17 families [29]). However, an apparently fixed host-group reversal onto an RTA-clade host, *Dictyna* spp. (Dictynidae), by a species of the clade II polysphinctine *Zatypota anomala* (Holmgren, 1860) [65] has taken place [67,75,140], but retaining typical *Zatypota* egg placement. This colonisation of an RTA-clade host presumably occurred because *Dictyna* inhabits aerial parts of plants (rather than being ground-dwelling) and produces webs similar to those of the theridiid (Araneoidea) hosts parasitised by most *Zatypota* species in similar habitats.

Any possible relationship between the position of the egg on the abdomen, the exact oviposition process, and the variable size and nature of the ventral expansion at the proximal end of the ovipositor among clade II taxa (weak in *Polysphincta* (Fig. 6D); apparently absent in *Oxyrrhexis* (Fig. 6A) and in two genera of completely unknown biology, *Chablisea* (Fig. 6B) and *Aravenator* (Fig. 6C)) is beyond the scope of this paper, but might be a worthwhile and revealing study.

## Conflict of interest

The authors declare that they have no competing or financial interests.

## Supplementary Material

Figure S1. Metasoma movement during the dorsal-press by *Brachyzapus nikkoensis*. Figure S2. Metasoma movement during the ventral-press by *Zatypota albicoxa*. The Supplementary Material is available at https://www.parasite-journal.org/10.1051/parasite/2018011/olm.
