# Supplementary figures and images for: The changing use of the ovipositor in host shifts by ichneumonid ectoparasitoids of spiders (Hymenoptera, Ichneumonidae, Pimplinae)
Source: Parasite. 2018 Mar 28;25:17. doi: 10.1051/parasite/2018011 (PMC5873220; doi:10.1051/parasite/2018011)

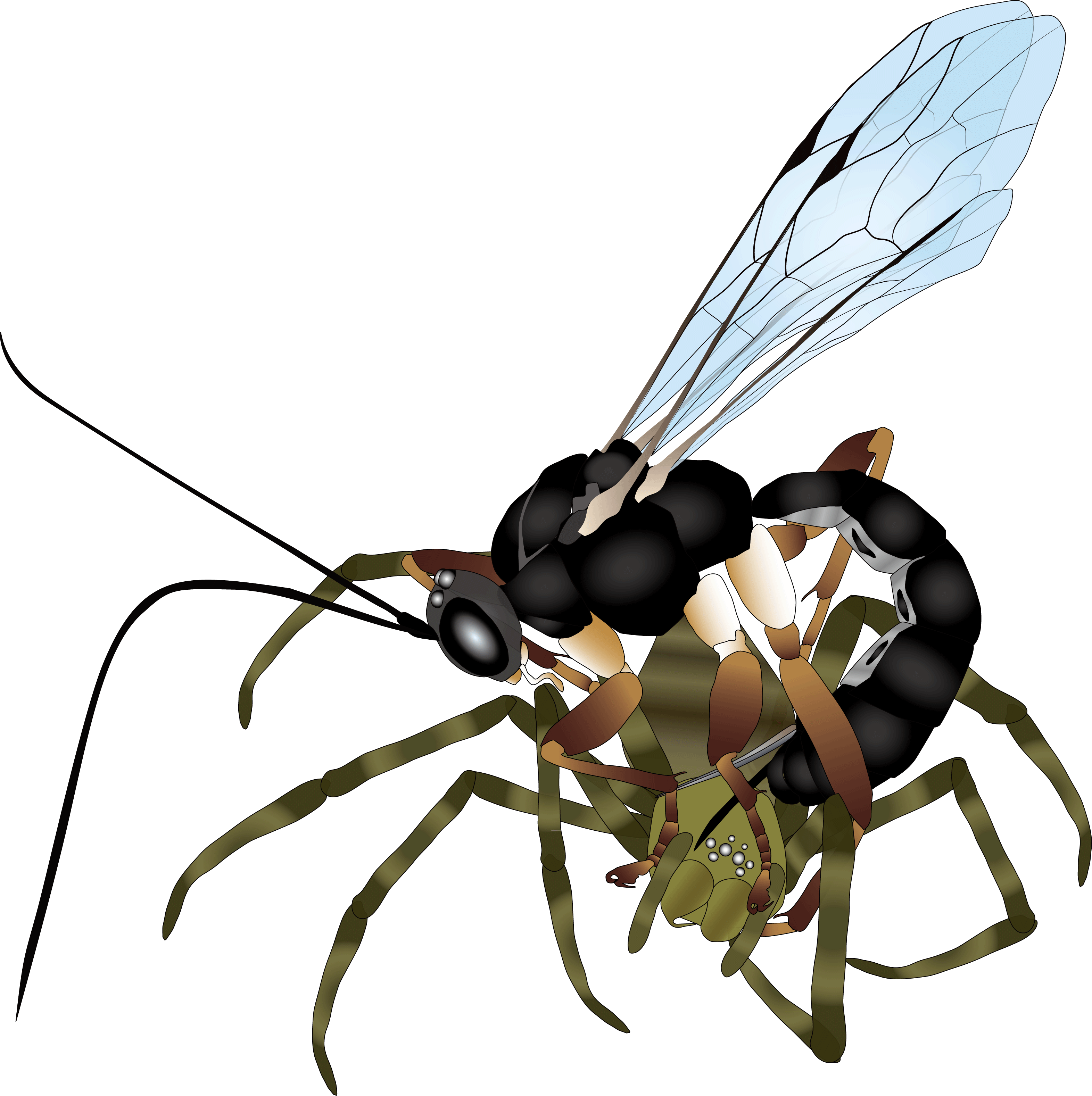

Supplement: Supplementary file 1 — Figure S1. Metasoma movement during the dorsal-press by Brachyzapus nikkoensis. [file parasite-25-17-s1.gif]

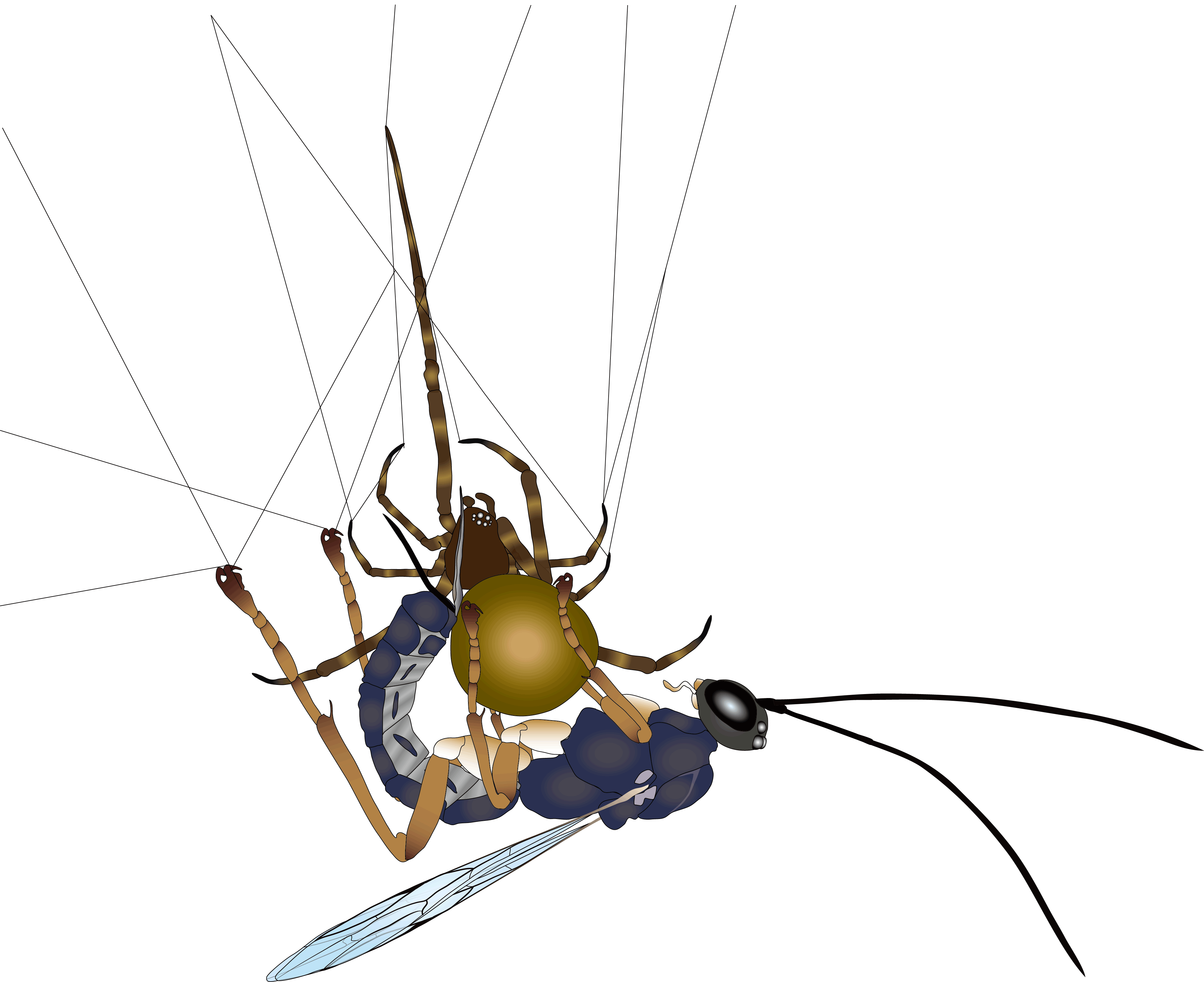

Supplement: Supplementary file 2 — Figure S2. Metasoma movement during the ventral-press by Zatypota albicoxa. [file parasite-25-17-s2.gif]
